# New Technique for S1 Nerve Root Transforaminal Percutaneous Fluoroscopically Guided Approach for Difficult Cases of Altered Anatomy

**DOI:** 10.3390/jcm14093126

**Published:** 2025-04-30

**Authors:** Łukasz Kubaszewski, Adam Druszcz, Wojciech Łabędź, Zofia Kubaszewska, Mikołaj Dąbrowski

**Affiliations:** 1 Department of Adult Spine Orthopedics, W. Dega Orthopedics and Rehabilitation Clinical Hospital, Poznan University of Medical Sciences, 28 Czerwca 1956 r. Str. 137/145, 61-545 Poznań, Poland; wlabedz23@gmail.com (W.Ł.); mdabrowski@ump.edu.pl (M.D.); 2 Neurosurgery Ward, Regional Specialist Hospital, Iwaszkiewicza Str. 5, 59-220 Legnica, Poland; drdruszcz@gmail.com; 3 Department of Medical and Surgical Sciences, Alma Mater Studiorum—Universita di Bologna, Via Zamboni, 33-40126 Bologna, Italy; zkubaszewska8@gmail.com

**Keywords:** lower back pain, S1 nerve, lumbar nerve root, fluoroscopic-guided localization

## Abstract

**Background:** S1 nerve roots are difficult to approach during percutaneous procedures for the diagnostic and treatment procedures of low back pain with radicular symptoms. This is harder in older patients with obscure anatomies, due to the low bone density with overimposing degenerative changes in the facets and deformations. The otherwise straightforward procedure for the lumbar nerve roots, placing the needle in the proximity of the S1 under fluoroscopic guidance, becomes quite a challenge. Case presentation: In the proposed technique, the initial target for the needle is the lower part of the S1 facet in the convergent trajectory of the needle. After achieving contact with the bone the tip of the needle is moved caudally as, in proximity, it reaches the dorsal foramina of the S1/S2 segment—this is named “wandering to the hole”. The convergent trajectory of the needle ensures the success of the procedure with a minimal risk of intravenous drug administration, which is characteristic for the suprapedicular technique. **Conclusions:** The proposed technique is straightforward and reproducible due to the combination of the understanding of the surgical and radiological anatomy of this region, in spite of degenerative changes in the spine.

## 1. Introduction

The sacral bone is one of the most complex bony structures when it comes to fluoroscopically guided percutaneous procedures. Great difficulties in performing percutaneous procedures are determined by the origin of the bone at the sacral vertebrae fusion, the connection to the pelvic ring and neighboring visceral structures. Therefore, during the procedure not an image but the experience of the performing doctor is the main component of success.

One of the most common percutaneous procedures performed in the sacrum is the reaching with a needle to the tip of the S1 nerve roots. It can be performed just for diagnostic infiltration, but also in the pulsed radiofrequency neuromodulation of the dorsal root ganglion (DRG).

The route to achieve the target is the dorsal foramina of the S1/S2 motion segment. However, it is difficult to visualize, especially in older individuals with coexisting degenerative changes or a high bone transparency in the course of osteoporosis. In such cases, the procedure can be quite uncomfortable for the patient when the random poking search of the foramina is performed.

On the other hand, the available described techniques are far from ideal, yielding inappropriate angles with a high risk of not reaching the target point.

One of the most common techniques is the subpedicular technique with an AP projection, as described in 2013 by Bogduk et al. [1,2]. Unfortunately, this technique may result in a high incidence of vascular puncture and unsatisfactory results [3], as outlined by the technique’s inventors. Furthermore, the presented results show that the placement of the needle is much more lateral than intended. An alternative approach requires a Scotty dog projection, as described by Fish et al. [4], which allows a more convergent needle placement compared to the AP techniques. Although, it is less handy in cases with advanced degenerative changes or in patients with a low bone mass density, where proper visualization is impossible.

As the salvage procedure, the multi-needle technique is performed. The multi-needle technique is based on the premise that multiple needles give the operator more information on the localization of the dorsal foramina and its orientation than the fluoroscopic image itself. It can be compared to stereoscopic vision and is considered as the countermeasure in cases of vascular puncture and dye administration [3].

Comparing both the AP and Scotty dog techniques, Kang et al. favor the latter in their review [5]. Recently the medial-to-lateral needle trajectory was published by Chapman et al. [6] and is designed to limit interactions with the sensitive neural structures. Though this technique was designed for the placement of a long-lasting implantable, DRG stimulation leads, and we lack further data on whether it is applicable for the drug injection.

According to Park et al. [7], it seems that for finding the dorsal foramina the ultrasound technique is much easier, although it also carries some drawbacks. The authors themselves admitted that, in some cases, fluoroscopy may be subsidiary to the localization of the needle, but compulsory to confirm the depth of the needle placement and verification of the vascular spread before the drug administration. Thus, only the US-verified needle placement technique is not sufficient.

Of course, the ultimate resource is the CT- or MRI-guided procedure, but due to the costs and organizational obstacles it is rarely performed. The MRI-controlled needle placement reaches a 97% success rate, but the literature review does not support the popularity of the method [8].

We have to conclude that the anatomical considerations of the S1/S2 foramina approach are quite poor in the literature. The visualization of the S1/S2 dorsal foramina in the majority of cases is impossible and an awareness of its anatomical variability leads to omitting it [9]. The meta-analysis of the topic shows that the scope of interest refers mainly to the lumbar foramina, rarely discussing the procedure referring to the S1 root [7,10,11].

A totally different technique is the preganglionic epidural steroid injection through the translateral recess approach described by Hwang et al. [12]. But this technique was described and is advised only for the L5/S1 segment due to its favorable anatomy.

Summarizing the above-mentioned techniques, this all makes the procedure demanding and frustrating to inexperienced practitioners, which sometimes is the reason for its abandonment.

The aim of this paper is to present a new, straightforward technique for performing the transforaminal approach to the S1 nerve root with the use of only fluoroscopic guidance, and also in occurrences of degenerative changes obscuring the anatomy or poor visualization due to low bone density.

## 2. Case Presentation and Technical Notes

### 2.1. Study Model

This study was conducted using a 3D anatomical model and 3D CT reconstruction to assess the transosseous approach through the S1/S2 dorsal foramen. The evaluation included both anatomical and radiological aspects in sagittal, coronal and axial projections.

### 2.2. Study Design

This study consisted of two main stages:

Anatomical and Imaging Analysis—A three-dimensional reconstruction based on CT images was performed to precisely visualize anatomical structures and assess the trajectory of the approach through the S1/S2 foramen.

Clinical Evaluation—The technique was applied in a single clinical case, monitoring the needle trajectory and its position relative to neural and bony structures.

### 2.3. Procedure

#### 2.3.1. Patient Positioning

The patient was placed in a prone position on a radiolucent table. Proper positioning, including hip flexion, was ensured to optimize the joint space visibility.

#### 2.3.2. Entry Point Identification

The skin entry point was localized at the tip of the L5 transverse process on the ipsilateral side. This positioning provided the correct coronal and sagittal plane inclination of the needle.

#### 2.3.3. Needle Insertion and Targeting

The primary target point was the inferior part of the L5/S1 facet joint or the caudal part of the S1 superior facet. A beveled needle (typically 18G–20G) was used to facilitate minor trajectory adjustments without the need for complete repositioning. Gentle tapping of the needle was performed to confirm contact with the joint capsule and later with the inferior part of the S1 facet.

#### 2.3.4. Advancing Toward the S1/S2 Foramen

Once the needle was positioned at the inferior part of the S1 facet, it was advanced caudally with controlled tapping. The transition from bone contact to a loss of resistance indicated entry into the dorsal S1/S2 foramen. The needle depth was confirmed using fluoroscopic lateral views, contrast injection or patient response to nerve stimulation.

#### 2.3.5. Safety Measures and Final Confirmation

A 1% lidocaine solution was used to create a hydrostatic cushion at the needle tip, reducing the risk of nerve root irritation. Tapping over the bone was employed to blunt the needle tip, further minimizing the risk of nerve injury. This methodology ensured a precise, reproducible needle placement while reducing the radiation exposure and optimizing procedural safety.

The main and most important feature of the technique is the fact that the dorsal foramina of the S1/S2 segment are located in the proximity of the L5/S1 facet joint, caudally in the line of the continuation of the axis of the inferior articular processes of the L5 vertebrae. This fact is commonly known among the spine surgeons, while the surgical preparation of this region may lead to bleeding due to puncturing the vessels emerging from that opening.

The entrance to the foramina is performed only in a plain AP view with no necessity to adjust it to the sacral bone inclination. There is no need to visualize the foramina or the other anatomy of the sacral bone. The main target is the facet joints. The patient is positioned prone on the radiolucent table. In order to simplify the procedure, the four following steps are introduced.

### 2.4. Entry Point

Due to the orientation of the S1/S2 dorsal canal, the needle trajectory has to be convergent in the coronal plane with inclination from the rostral (with entry point) to the caudal (for the final target) in the sagittal plane.

**Figure 1:**
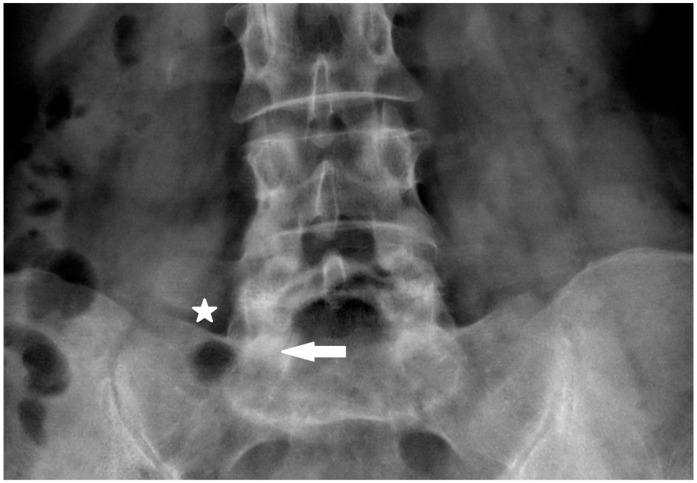
Skin entry point superior edge or slightly above sacral ala ⅓ lateral part of distance between lateral margin of L5/S1 facet and superior brink of sacroiliac joint (asterisk—skin entry point, arrow—target point of first bone contact).

Therefore, the skin entry point is localized at the tip of the L5 transverse process on the same side (Figure 1). This yields the proper inclination of the needle in both planes, the coronal and sagittal.

### 2.5. Primary Target Point

**Figure 2:**
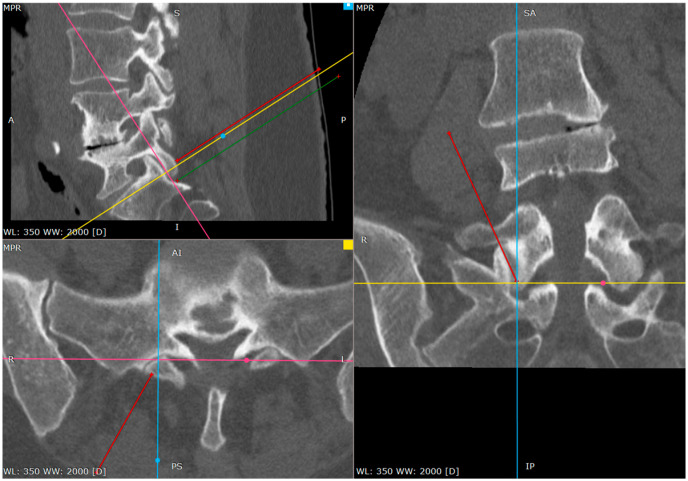
The primary target point—the inferior part of the facet (a CT tri-plane reconstruction).

**Figure 3:**
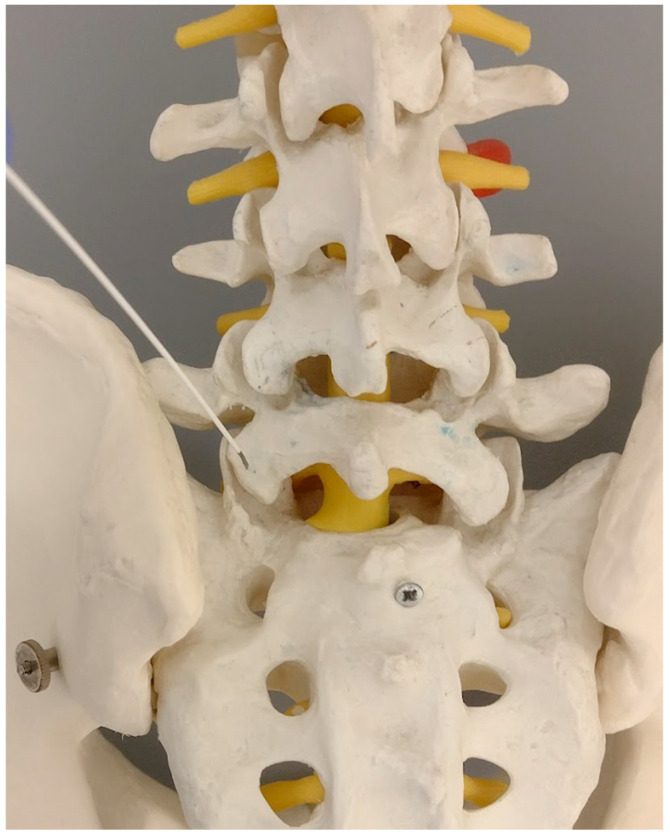
The primary target point—an inferior part of the facet (a model).

The primary target point for the needle introduction is the lower part of the facet joint or the caudal part of the articular surface of the S1 superior facet (Figure 2 and Figure 3).

It is safer to aim at the lower part of the facet joint, because it is an easily identifiable structure in any anatomical variation.

The use of a needle with a tilted end is the most convenient for the procedure. It enables the trajectory modification without the need for withdraw, while twisting the tip in the desired direction (allows for the slight modification of the tip orientation while the needle is advanced). The size of the needle is usually 20G through 18G, depending on the type of the procedure, although we prefer the larger size as it provides better control over the direction.

The trajectory is similar as in the approach of the lateral ramus of the posterior branch innervating the caudal part of the facet joint. Actually, a neurolesion of this branch could be performed as the first part of the procedure.

The needle is advanced toward the lower part of the L5/S1 facet joint. After the bony tissue’s contact with the gentle tapping of the needle the “soft over bony structure” of the capsule may be identified. This tapping can be utilized for the gentle anesthesia of the region, with a small amount of lidocaine, as further tapping of the bone will be performed.

### 2.6. Contact with Articular Surface

With this tapping motion (under anesthesia) we move caudally. With this, the needle slips over the inferior tip of the inferior facet of the L5. There is a significant change in the sensation the operator receives from tapping.

The “soft over bony structure” of the joint capsule changes to “pure bone” contact. This is the lower surface of the superior articular process of the S1. In healthy structures the consistency of the cartilage can be perceived. Actually, at this stage the tip of the needle is exactly in the joint space in its most spacious part. With the proper positioning of the patient—hip flexion—the joint space increases and drugs of choice can be administered locally.

**Figure 4:**
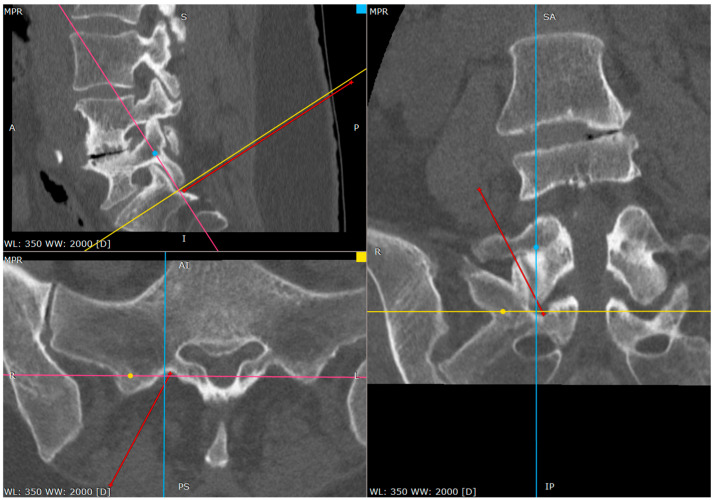
Needle against inferior part of S1 facet surface (CT tri-plane reconstruction).

**Figure 5:**
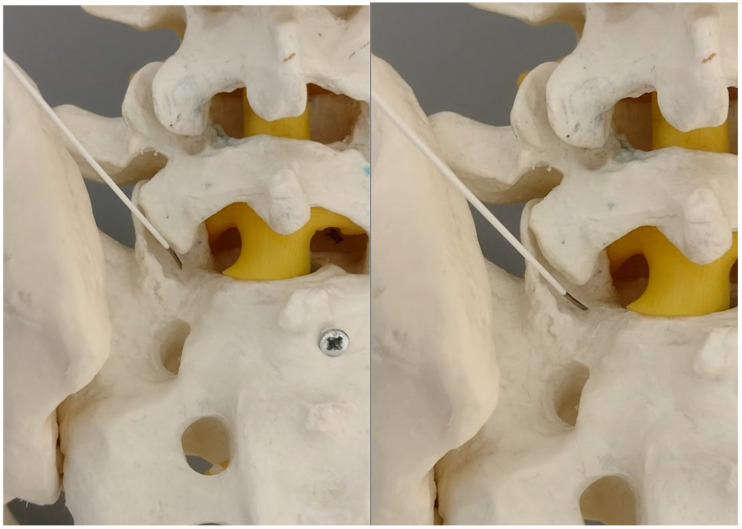
The needle against the inferior part of the S1 facet surface (model). On the right side—twisting the bended tip of the needle points against the medial rim of the S1 facet.

As the needle is placed against the S1 facet it is the secure spot (Figure 4 and Figure 5).

The S1 facet has a cradle-like shape in the transverse plane, so there is no risk of spinal canal violation, as long as it is lateral to the medial border of the S1 inferior facet in the AP view, which is being used from the beginning of the procedure.

The operator can check it by tapping with the tip of the needle toward the midline. There is a sensation of the upward slope; the sudden advancement of the needle with no bone contact necessitates the verification of the needle tip.

### 2.7. Wandering to the Hole

**Figure 6:**
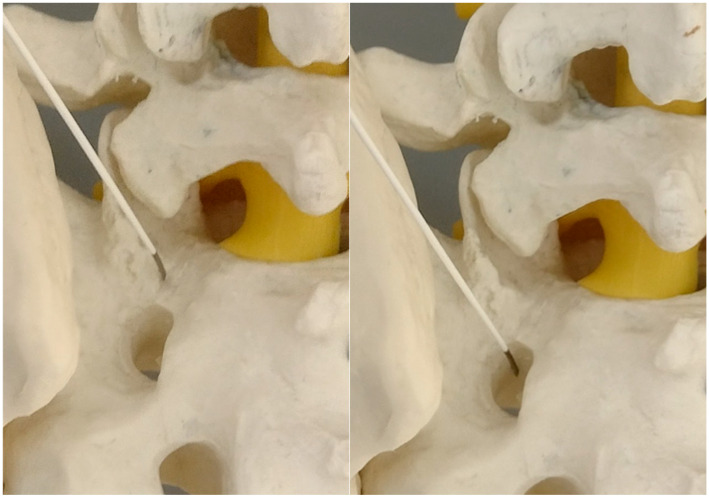
The downward movement of the needle toward the foramina.

At this phase we start to wander to the caudal direction with the tip of the needle by tapping, moving the tip with a gentle reversing–advancing movement, as well as the twisting of the bended tip (Figure 6).

In a short distance the needle loses the bony contact as it reaches the dorsal foramen of the S1/S2 segment.

It is beneficial to remember the level of it. If we do not perform it by then, the needle can be reversed and placed on the brink of the opening, to give an idea of what the depth is at the start of the opening.

From this point we can gently advance the needle 2–3 cm before we confirm the depth of the needle on the lateral view.

**Figure 7:**
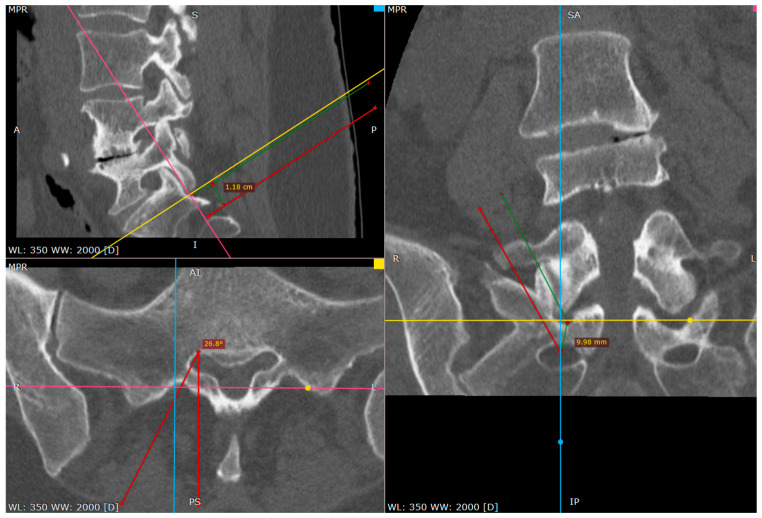
Distance between inferior facet surface and dorsal foramina (CT tri-plane reconstruction).

**Figure 8:**
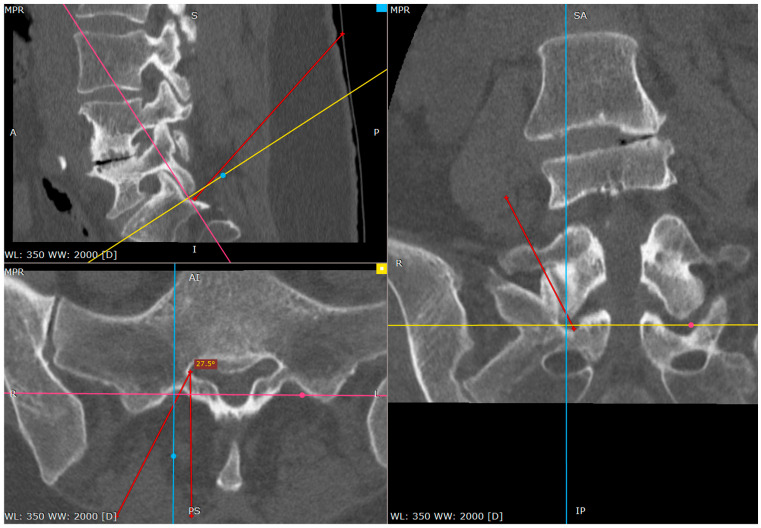
Approach angle and needle trajectory to facet with intent to approach S1 root (CT tri-plane reconstruction).

**Figure 9:**
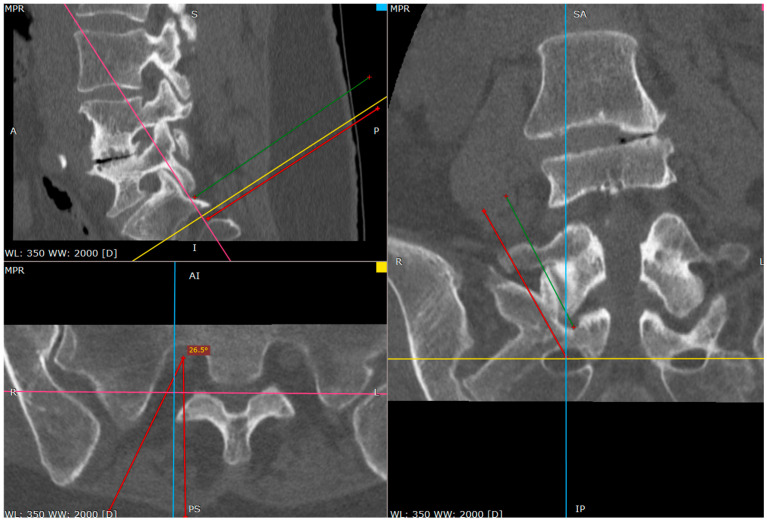
Approach angle and needle trajectory to S1 root (CT tri-plane reconstruction).

The proper placement of the tip can be confirmed with the contrast administration, fluoroscopy verification or clinical response with the S1 root distribution sensation after the tip of the needle contacts the S1 sheath. Therefore, at this part one should be cautious. We usually use the 1% lidocaine solution to produce the hydrostatic pressure cushion at the tip of the needle, while advancing the needle, as well as a larger needle that poses less risk of penetrating the root sheath. Also, the tapping activity over the bone blunts the tip of the needle, decreasing the risk of root injury (Figure 7, Figure 8 and Figure 9).

The postoperative course was uneventful, with no reported complications. The patient experienced clinical improvement in terms of pain reduction and functional mobility. The radiation exposure during the procedure was within acceptable limits and did not exceed standard intraoperative thresholds for this type of intervention.

### 2.8. Preparation of the Manuscript

We declare that during the preparation of this manuscript, the author(s) used Chabot App (https://chat.chatbotapp.ai/chats/) for the purposes of text editing in the Case Presentation and Technical Notes Section 2.1, Section 2.2 and Section 2.3 and finding additional citations in the Discussion section. The authors have reviewed and edited the output and take full responsibility for the content of this publication.

## 3. Discussion

The main asset of the procedure is that it utilizes the major unequivocal anatomical landmarks in this region. This procedure may limit radiation exposure. Only in the first and second step is fluoroscopy essential for the foramina localization. The sliding over the facet joint, contact with its cartilage and later finding the opening, in majority of the cases, can be performed without radiology. Later, only the localization of the needle tip in relation to the S1 root may necessitate a lateral fluoroscopic confirmation to avoid painful mechanical root irritation. With the help of the neurostimulation equipment the proximity of the root can be determined before the mechanical contact. The convergent trajectory mitigates the risk of entering the presacral region, because the tip of the needle is directed towards the medial wall of the caudal canal of the S1/S2 motion segment. Additionally, neurostimulation can enhance safety by determining the root proximity before the direct needle contact, which is an approach that has been widely recognized in percutaneous spinal interventions [13,14].

A key feature of this approach is the convergent trajectory of the needle, which reduces the risk of violating the presacral region. The needle tip is directed toward the medial wall of the caudal canal at the S1/S2 motion segment, which ensures greater procedural safety compared to more traditional transforaminal approaches. Studies on fluoroscopy-guided sacral injections have shown that a precise trajectory significantly decreases the likelihood of vascular injury and improper drug distribution [15,16].

The technique is also unaffected by major anatomical variations, common in the advanced degeneration or poor radiological visualization in low bone density cases [17]. The primary target point is the L5/S1 facet which can be visualized in all cases. The only modality is that in subluxated joints the inferior joint surface cannot be detected by the “touch of the needle”, and the distance between the inferior tip of the L5 process and the dorsal foramina can be significantly decreased, but in our practice this is very rare.

Unlike the almost sagittal trajectory of pedicle-based techniques, which carry a higher risk of bypassing the nerve root or inadvertently puncturing vascular structures, this approach allows for the controlled needle advancement while maintaining the proximity to the nerve without unnecessary risk [18,19,20].

Recent anatomical studies provide valuable insights into the application of our proposed fluoroscopically guided transforaminal approach to the S1 nerve root, particularly in patients with an altered sacral anatomy. Suzuki et al. conducted an analysis of the S1 neural foramen using three-dimensional computed tomography (3D CT), which allowed for a better understanding of its anatomical variations and optimal fluoroscopic angles for the needle placement. Their study suggests that the precise localization of the S1 neural foramen and the determination of safe puncture depths can significantly improve the procedural accuracy and safety in S1 nerve root blocks [21].

A previous study demonstrated a high success rate (92.9%) for the fluoroscopically guided S1 nerve root approach [7]. The identification of the S1 foramen has traditionally been considered highly dependent on both the operator’s expertise and patient-specific anatomical variations.

This study has several limitations. First, it is a single-center study, which may restrict the generalizability of our findings. Future multi-center studies are necessary to validate our results and assess the broader applicability. Second, while we acknowledge the importance of operator experience, our study does not provide a detailed analysis of how expertise influences success rates. Additional research should focus on whether this technique can be reliably performed by less experienced clinicians or requires advanced training. Third, anatomical variations, such as lumbo-sacralization, may affect the accuracy and feasibility of this technique. Pre-procedural imaging, including CT or MRI, may be necessary to assess the anatomical suitability in such cases. Additionally, although we have compared our results with previous studies, direct head-to-head comparisons with alternative techniques would provide a more robust evaluation. Finally, the sample size in our study may not be sufficient to capture all factors affecting procedural success and complications. Future studies with larger cohorts are needed to strengthen our conclusions.

By integrating these findings with previously established evidence on sacral and lumbar spine interventions, this method represents a safe and effective alternative for accessing the S1/S2 region while minimizing radiation exposure, anatomical variability and potential complications.

## 4. Conclusions

Percutaneous pain treatment procedures are rarely performed by professionals that have daily practice with the surgical anatomy. Therefore, the majority of training in this area is based solely on the radiological anatomy. The invention of the technique was possible by fusing the surgical and radiological anatomy knowledge. It engages partly the “feeling” of the local anatomy, similar to palpation, not only relying on fluoroscopic images. We hope the community performing this procedure will find our proposal helpful. Further studies and clinical experience will help refine its application and validate its broader clinical utility.

## Data Availability

The original contributions presented in this study are included in the article. Further inquiries can be directed to the corresponding author.
